# Effectiveness and Healthcare Cost of Adding Trastuzumab to Standard Chemotherapy for First-Line Treatment of Metastatic Gastric Cancer: A Population-Based Cohort Study

**DOI:** 10.3390/cancers12061691

**Published:** 2020-06-25

**Authors:** Matteo Franchi, Roberta Tritto, Lorena Torroni, Chiara Reno, Carlo La Vecchia, Giovanni Corrao

**Affiliations:** 1 National Centre for Healthcare Research and Pharmacoepidemiology, 20126 Milan, Italy; roberta.tritto@unimib.it (R.T.); giovanni.corrao@unimib.it (G.C.); 2 Laboratory of Healthcare Research & Pharmacoepidemiology, Department of Statistics and Quantitative Methods, University of Milano-Bicocca, 20126 Milan, Italy; 3 Unit of Epidemiology and Medical Statistics, Department of Diagnostics and Public Health, Università degli studi di Verona, 37134 Verona, Italy; lorena.torroni@univr.it; 4 Department of Biomedical and Neuromotor Sciences, Alma Mater Studiorum University of Bologna, 40126 Bologna, Italy; chiara.reno@studio.unibo.it; 5 Department of Clinical Sciences and Community Health, Università degli Studi di Milano, 20122 Milano, Italy; carlo.lavecchia@unimi.it

**Keywords:** gastric cancer, trastuzumab, overall survival, real-word evidence, cost-effectiveness

## Abstract

A randomized clinical trial showed that trastuzumab, added to traditional chemotherapy, significantly improved overall survival in human epidermal growth factor receptor 2 (HER2)-overexpressing metastatic gastric cancer patients. This population-based study aimed at evaluating both the clinical and economic impact of trastuzumab in a real-world setting. By using the healthcare utilization databases of Lombardy, Italy, a cohort of patients newly diagnosed with metastatic gastric cancer during the period 2011–2016 was selected. Among these, patients initially treated with either trastuzumab-based chemotherapy or standard chemotherapy alone were followed up until death, migration in other regions or June 2018. Overall survival and average cumulative costs were estimated and compared between the two treatment arms. Among the 1198 metastatic gastric cancer patients who started therapy within six months after metastasis detection, 87 were initially treated with trastuzumab-based chemotherapy and 1111 with standard chemotherapy. Median overall survival and restricted mean survival were 10.2 and 7.4 months, and 14.9 and 11.4 months, respectively, in the two treatment arms. The adjusted hazard ratio of death was 0.73 (95% CI 0.57–0.93). The average per capita cumulative healthcare costs were, respectively, EUR 39,337 and 26,504, corresponding to an incremental cost-effectiveness ratio of EUR 43,998 for each year of survival gained. Our study shows that adding trastuzumab to conventional chemotherapy is effective and cost-effective.

## 1. Introduction

Trastuzumab (Herceptin^®^, Roche, Basel, Switzerland) is the first humanized monoclonal antibody against human epidermal growth factor receptor 2 (HER2), approved late in 2010 by the U.S. Food and Drug Administration (FDA) in patients with HER2-overexpressing metastatic adenocarcinoma of the stomach or gastro-oesophageal junction (gastric cancer) who have not received prior anti-cancer treatment for the metastatic disease [1]. This approval was granted after the pivotal phase III ToGA trial showed that, among 584 patients with previously untreated HER2-overexpressing advanced gastric cancer, the addition of trastuzumab to standard chemotherapy prolonged median overall survival (OS) from 11.1 to 13.8 months, as compared to patients treated with chemotherapy alone (*p* = 0.0046) [2]. Based on these results, Italian guidelines support the recommendation for trastuzumab treatment in HER2-overexpressing metastatic gastric cancer [3]. However, no further evidence is available, neither in the experimental setting, nor in the post-marketing real-world setting, for evaluating the added value of trastuzumab in gastric cancer patients. 

Based on these premises, we carried out a population-based study for assessing both the clinical impact on overall survival and the economic impact on the National Health Service (NHS) of the treatment with trastuzumab-based chemotherapy, as compared to standard chemotherapy alone, in a real-world cohort of metastatic gastric cancer patients.

## 2. Results

### 2.1. Patients

**Figure 1:**
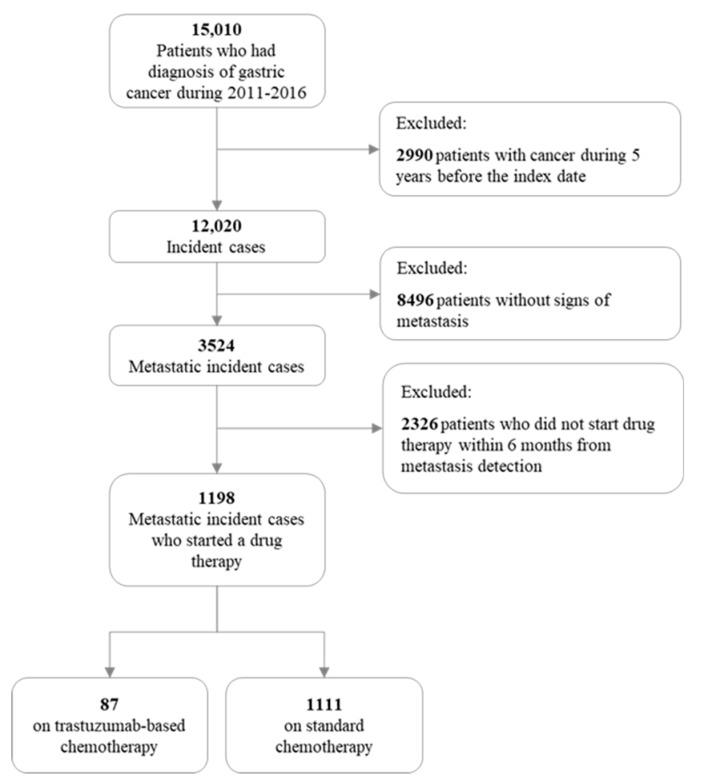
Flow chart of the inclusion and exclusion criteria in the final cohort study.

During the period 2011–2016, 15,010 patients with a diagnosis of gastric cancer were identified. Of these, 12,020 were incident patients, and for 3524 (29.3%) of them metastasis was detected at the time of their gastric cancer diagnosis (or within six months). Finally, of the 1198 patients who started chemotherapy within six months from metastasis detection, 87 (7.3%) received trastuzumab-based chemotherapy and 1111 (92.7%) standard chemotherapy (Figure 1). Their median age was 67–66 years, and 74% and 64% of them were men. There was no evidence of between-arm difference for sex, age, year of diagnosis, surgery or clinical profile (Table 1).

### 2.2. Survival Comparisons

**Figure 2:**
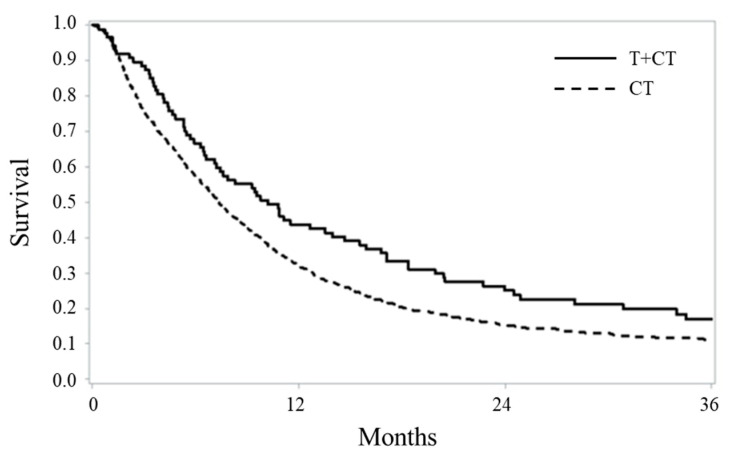
Kaplan–Meier estimates of overall survival in 87 and 1111 metastatic gastric cancer patients treated, respectively, with trastuzumab-based chemotherapy (T + CT) and standard chemotherapy (CT) alone.

After a median follow-up of 7.4 months, 76 (87.4%) deaths occurred among patients on trastuzumab and 994 (89.5%) in the standard arm. The proportions of survivors after 1, 2 and 3 years from starting therapy were 44%, 25% and 17% among patients on trastuzumab and 32%, 15% and 11% among those on standard therapy. The median OSs, respectively, were 10.2 months and 7.4 months (log-rank test, *p* = 0.0147) (Figure 2), and the restricted mean survivals were 14.9 and 11.4 months.

The unadjusted and adjusted hazard ratios (HRs) of death associated with trastuzumab therapy, respectively, were 0.74 (95% CI: 0.58 to 0.95) and 0.73 (0.57 to 0.93). The independent predictors of OS were gender and surgery, i.e., women and patients that underwent surgery had lower mortality (Table 2).

### 2.3. Healthcare Cost and Cost-Effectiveness Profile

On average, the healthcare costs along the entire period of follow-up of a cohort member on trastuzumab and standard chemotherapy, respectively, were EUR 39,337 and 26,504. Costs for inpatient and outpatient services and medicaments, respectively, were EUR 14,010, 6594 and 18,733 for patients on trastuzumab, and EUR 17,058, 4024 and 5422 for those on standard chemotherapy. 

**Figure 3:**
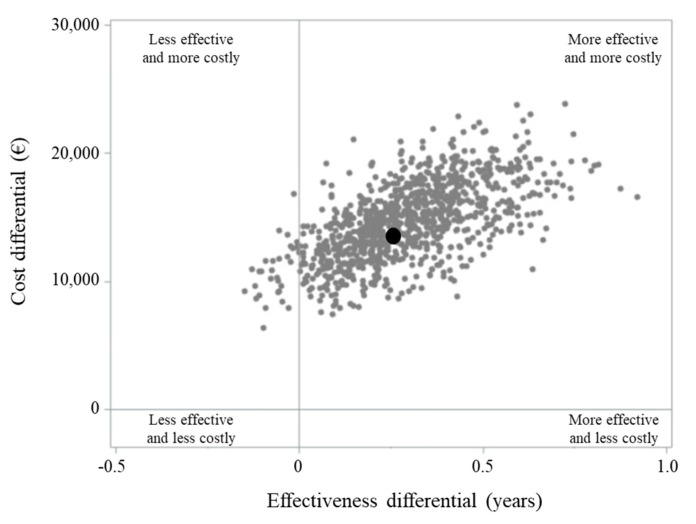
Incremental cost-effectiveness ratio (ICER) scatterplot comparing metastatic gastric cancer patients treated, respectively, with trastuzumab-based chemotherapy (T + CT) and standard chemotherapy (CT) alone.

The incremental cost-effectiveness ratio (ICER) value indicated an average cost of EUR 43,998 for each year of life gained by the treatment with trastuzumab. The ICER value was suggestive of clinical effectiveness (longer survival for patients on trastuzumab), constrained to higher healthcare costs in 97% of the 1000 bootstrap replications (Figure 3).

### 2.4. Sensitivity Analyses

In total, 115 and 1083 patients who were, respectively, classified into the trastuzumab and standard chemotherapy arm by lengthening the time-span for admitting a cohort member into the trastuzumab arm from 21 days to 42 days, had a median OS of 10.2 and 7.3 months (*p* = 0.0159), with an adjusted HR of 0.74 (0.59 to 0.91). The favorable role of trastuzumab was confirmed when accounting for the time-varying exposure to therapies (HR 0.73 (0.57 to 0.93)). When accounting for HER2 overexpression, reduced mortality ranging from 49% to 29% and ICER values ranging from EUR 16,176 to 22,984 were obtained, assuming the prevalence of HER2+ among patients on standard chemotherapy to be 10% and 90%, respectively. Finally, the median OS from the propensity score (PS) matched design became 10.2 and 7.4 months (*p* = 0.0157), with an HR of 0.66 (0.49 to 0.90).

## 3. Discussion

The present study showed that, in a population-based cohort of gastric cancer patients already metastatic at diagnosis, those treated with trastuzumab-based chemotherapy had a significant 27% reduction in the risk of death compared with those on chemotherapy alone. To our knowledge, this represents the first study investigating the effectiveness of trastuzumab as a first-line therapy of metastatic gastric carcinoma in a real-life setting. Our study supports decision makers, informing them that the additional healthcare cost that a payer should bear by adding trastuzumab to conventional chemotherapy was EUR 44 thousand per year-of-life gained, a cost lower than the willingness-to-pay thresholds ranging from EUR 50 thousand to 100 thousand per year-of-life gained that are frequently adopted from western countries [4,5].

Our results are consistent with those reported by the ToGA trial. In our study, patients treated with trastuzumab-based chemotherapy experienced a gain in median OS of 2.8 months (10.2 vs. 7.4 months) as compared to those treated with standard therapy alone. The corresponding figure in the randomized clinical trials (RCT) was 2.7 months (13.8 vs. 11.1 months) [2]. The RCT showed median OS in the two treatment arms to be higher than those observed in our study. This may be explained by the characteristics of patients included in the clinical trial that, because of specific inclusion and exclusion criteria, are expected to have more favorable clinical profiles than patients observed in clinical practice [6]. Indeed, patients included in the RCT had a median age of 59 years, good daily living abilities (performance status), acceptable physiologic status, and did not suffer from cardiovascular diseases, malabsorption syndrome, gastrointestinal bleeding, or brain metastases [2]. In contrast, patients included in our study had a median age of 67 years, and were not submitted to any exclusion criteria as compared to the ToGA trial.

In our cohort, 7.3% of patients were treated with trastuzumab. Although the ToGA trial showed that metastatic gastric cancer HER2+ patients do benefit from the treatment with trastuzumab, it is not surprising that only a low percentage of the cohort members were eligible to be treated with trastuzumab. Indeed, the frequency of HER2+ (i.e., patients eligible to be treated with trastuzumab) varies widely, with values ranging from 4.4% to 53.4%. This heterogeneity may be explained, at least in part, by the different methods used for assessing HER2. In particular, HER2 status is mainly assessed by immunohistochemistry (IHC) or in situ hybridization (ISH) assays. The fluorescent in situ hybridization (FISH), that is considered the gold standard, is generally used only for unclear cases, because of its higher cost, time consumption and the need for a fluorescence microscope [7].

The HER2 prognostic values on overall survival are controversial. However, although some small-case studies found an (non-significant) association with better overall survival [8,9,10,11], several meta-analyses showed that HER2 is a negative prognostic factor, with more aggressive biological behavior and higher frequencies of recurrence in HER2-overexpressed tumors [12,13,14,15,16]. In particular, the meta-analysis by Gu et al. was the only one showing no association, the summarized HR being 0.97 (0.84 to 1.12) [16]. The other meta-analytic estimates vary from 1.47 (1.09 to 1.98) [14] to 1.66 (1.35 to 2.02) [12]. By admitting a positive association between HER2-overexpressing and OS, and assuming that trastuzumab was administered only to HER2+ patients (as indicated by the national guidelines), the observed clinical benefit of trastuzumab treatment (i.e., a 27% reduction in mortality) is expected to be even stronger than those observed in our study. Indeed, by assuming that HER2+ patients have a 1.5-fold increased risk of death than HER2 negative patients, adjusted mortality reduction associated with trastuzumab fell to 29%, or even 49% by assuming a prevalence of HER2+ patients on standard chemotherapy of 90% and 10%, respectively.

The cost-effectiveness of trastuzumab in metastatic gastric cancer is controversial [17,18]. A cost-effectiveness analysis based on the ToGA trial showed that the ICER per year-of-life gained was EUR 81 thousand in all HER2+ cancer patients, while it decreased to EUR 60 thousand and EUR 39 thousand in specific HER2 patient subgroups [17]. Although our findings, as well as those based on the ToGA trial, suggest that trastuzumab is cost-effective for the therapy of HER2+ metastatic gastric cancer, inconsistent findings had been reported by a Chinese investigation [18]. No other cost-effectiveness analyses are available, to our knowledge, in the scientific literature.

The present study has several strengths. First, the target population from which we selected the study cohort was representative of routine clinical practice in the Lombardy Region. Indeed, all of the beneficiaries of the NHS hospitalized with a diagnostic code of gastric cancer and a concurrent (i.e., during the following six months) code of distant metastasis during the recruitment period were included in the study, with no restrictions on age and concomitant diseases. Second, to the best of our knowledge, this is the first study performed so far evaluating the post-marketing clinical impact of trastuzumab in metastatic gastric cancer patients. Third, other than the clinical impact, our study also evaluated the economic impact of trastuzumab in a real-world setting, adding evidence to the few already available. Fourth, several sensitivity analyses confirmed the robustness of the main findings.

A main limitation of the study is the lack of information on HER2 status and the paucity of additional clinical and biological data, as well as information on therapeutic patterns (e.g., first-line monotherapy or combination therapy with two or more chemotherapy drugs). Indeed, since patients were not randomly allocated to either trastuzumab therapy or standard therapy, the results may be affected by confounding. That is, the association with OS observed in this study might have been generated by factors influencing both the chemotherapeutic regimen and death. The potential effect of the HER2 status was widely taken into account in one of the four sensitivity analyses performed, and factors such as ethnicity and socioeconomic status can be confidently ruled out because the Lombardy population is largely Caucasian and has universal access to free-of-pay cancer care ensured by the NHS. Finally, our cohort was also matched by propensity scores, minimizing, thus, measurable confounding. Other unmeasured factors, such as smoking status, dietary patterns and helicobacter pylori infection might affect our conclusions.

A major weakness of our cost-effectiveness estimates is that they reflect the burden in clinical practice in the investigated setting (Lombardy, in the years 2011–2016), rather than supply generalizable estimates. We took the uncertainty caused by the unknown HER2 status of the included patients into account, by simulating what would have happened if, in clinical practice, few (10%) or even almost all (90%) the patients on conventional chemotherapy had been HER2+. Consequently, even in the absence of generalizable evidence, we could observe the ICER ranging from EUR 16,176 to 22,984, suggesting that trastuzumab is cost-effective for any scenario we can imagine in clinical practice.

## 4. Materials and Methods 

### 4.1. Setting

Data were retrieved from the healthcare utilization databases of Lombardy, a region of Italy that accounts for approximately 16% (almost ten million) of its population. The Italian National Health Service (NHS) provides universal and mostly free of charge healthcare services, including those for cancer care. In Lombardy, an automated system of databases to collect a variety of information is available. Details of the healthcare utilization databases of the Lombardy Region and of their use in the field of cancer have been reported elsewhere [19]. Specific diagnostic and therapeutic codes used for the current study are given in the Supplementary material (Table S1).

### 4.2. Cohort Selection and Follow-Up

The beneficiaries of the National Health Service (NHS) who were residents in Lombardy, were aged 18 years or older, who during the years 2011 to 2016 had at least one hospital admission for gastric cancer (the admission date of the first hospitalization being defined as “index date”), and who had signs of metastasis within six months from the index date, were included in the study cohort. Among these, we excluded those who already had a hospital admission and/or a drug prescription suggestive of cancer in the five years before the index date, and those who did not start chemotherapy within six months from the date of metastasis.

Patients accumulated person-time of follow-up from the date of starting chemotherapy until the earliest date among death (i.e., the primary outcome), migration, 30 June 30, 2018, or 3 years after starting therapy (being too few patients who survived longer).

### 4.3. First-Line Therapy

Patients were classified in the trastuzumab or standard chemotherapy arm according to whether they did or did not receive trastuzumab within 21 days (that is the duration of a chemotherapy cycle) from starting therapy.

### 4.4. Baseline Characteristics

Covariates included sex, age at diagnosis and year of index date. Patients were classified according to whether they underwent surgery between index or starting therapy dates. In addition, the Multisource Comorbidity Score (MCS) [20], a score recently developed and validated in Italy, was measured for assessing the clinical profile of each cohort member. For the purpose of the present study, the weights of the conditions that contribute to the score were recalculated by considering the cohort of cancer patients, rather than the general population—as was the case in the original version of the MCS [21].

### 4.5. Statistical Analyses

Between-arm differences in baseline characteristics were assessed using the Chi-Square test statistics, or its version for trend.

Overall survival (OS) was estimated by using the Kaplan–Meier estimator, and the Log–Rank test was used for testing between-arm differences. The association between the use of trastuzumab and risk of death was estimated by means of a multivariate Cox proportional hazard model. Estimates were expressed as hazard ratio (HR), along with 95% confidence intervals (CIs), adjusted for the above-listed covariates.

Between-arm differences in healthcare costs were also considered. For each patient, overall healthcare costs from the NHS perspective were calculated by the amount that the Regional Health Authority reimbursed to providers of drug therapies and inpatient and outpatient services (not only for cancer care). Cumulative healthcare costs were calculated by means of the Bang and Tsiatis estimator [22], a method that takes into account censored cost data.

The incremental cost-effectiveness ratio (ICER) was measured by dividing the between-arm differences in healthcare costs and health-related outcomes. The health-related outcome was measured by the restricted mean survival time (RMST), calculated as the area under the Kaplan–Meier curve [23], representing the average survival time experienced by each cohort member [24,25]. Thus, the ICER represents the additional healthcare expenditure for gaining one year of life due to the treatment with trastuzumab. The non-parametric bootstrap method based on 1000 re-samples [26] was used to explore the uncertainty in the cost-effectiveness estimates [27].

### 4.6. Sensitivity Analyses

Four sensitivity analyses were performed in order to assess the robustness of the main results. First, because of the arbitrariness in the choice of time-window defining the first-line therapy duration (21 days), we verified whether the main results changed after lengthening the time-window at 42 days.

Second, since the patients belonging to the standard chemotherapy arm may have switched to trastuzumab during follow-up (i.e., after 21 days from the start date of treatment), the exposure of interest was considered in the Cox model as a time-dependent variable. 

Third, trastuzumab should be exclusively used in HER2-overexpressing metastatic gastric cancer patients according to national guidelines [28]. However, because information on HER2 was not available in the regional database, as an unknown portion of HER2-overexpressing patients is unlikely to be treated with trastuzumab in clinical practice, and knowing the controversial prognostic significance of HER2 expression in patients with gastric cancer [12,13,14,15,16], between-arm difference in the clinical outcome observed from the main analysis might be due to the HER2-overexpressing rather than because of the therapeutic effect (unmeasured confounding). Accordingly, we tried to detect the extension of the confounding required to fully account for the exposure–outcome association (i.e., for moving the observed point estimate to null) [29]. With this aim, we (i) assumed all patients on trastuzumab to be HER2 overexpressed (henceforth HER2+ as opposed to HER2-), (ii) set the prevalence of HER2+ on standard chemotherapy to vary from 10% to 90%, and (iii) set the mortality of HER2+ to be 1.5-fold higher than those that were HER2- [13].

Finally, other than HER2-overexpression, other unmeasured features may explain the between-arm difference in the clinical outcome, e.g., those linked with the choice of treating a patient with either trastuzumab or standard chemotherapy. The propensity score (PS) methodology [30] was used to take into account unmeasured residual confounding. A multivariable logistic regression was used to model the probability of being treated with trastuzumab (i.e., the PS), given a set of covariates. The latter was those above-listed as baseline characteristics, including those used for computing MCS. Each cohort patient belonging to the trastuzumab arm was matched with (up to) four patients randomly selected from those on the standard chemotherapy arm who had the same value of PS as the corresponding index case, tolerating an absolute difference of 0.01. 

All analyses were performed using SAS 9.4 (Cary, NC, USA). A 2-sided *p*-value of 0.05 or less was considered significant.

### 4.7. Considerations on Sample Size

From preliminary data based on this setting, the expected number of metastatic gastric patients treated with first-line trastuzumab or standard chemotherapy is about 100 and 1100, respectively. By considering a one-tail first type error of 0.05, a median OS in the standard arm of 11.1 months [2] and a 26% reduction in the risk of death in patients treated with trastuzumab [2], the study will have a statistical power of 85.7% to detect a hazard ratio of death equal or less than 0.74.

### 4.8. Ethical Issues

The Ethical Committee of the University of Milano-Bicocca approved the protocol No. 506, entitled “Valutazione dell’utilizzo dei farmaci biologici nel paziente oncologico: progetto FABIO (Farmaci Biologici in Oncologia)” and established that the study (i) was exempt from informed consent (according to the General Authorization for the Processing of Personal Data for Scientific Research Purposes issued by the Italian Privacy Authority on 15 December 2016 [1]. (ii) provides sufficient guarantees of individual records anonymity, and (iii) was designed according to the quality standards of good practice of observational research based on secondary data.

## 5. Conclusions

In conclusion, our study offers evidence that adding trastuzumab to conventional chemotherapy in the first-line treatment of HER2 over-expressed metastatic gastric cancer is effective and cost-effective, and should be therefore adopted as a therapeutic strategy to improve the survival of these patients. Our study helps to inform such decisions, and highlights the need for further similar analyses in other countries.

## Figures and Tables

**Table 1:** Baseline characteristics of 87 metastatic gastric cancer patients treated with trastuzumab-based chemotherapy (T + CT) and 1111 patients treated with standard chemotherapy (CT).

Characteristic	Treatment Group		*p*-Value
	T + CT (*N* = 87)	CT (*N* = 1111)	
Age at diagnosis			
<55	10 (11.5)	204 (18.4)	0.140 *
55–64	31 (35.6)	285 (25.7)	
65–74	31 (35.6)	371 (33.4)	
75	15 (17.2)	251 (22.6)	
Median	67	66	
Sex			
Women	23 (26.4)	402 (36.2)	0.067
Men	64 (73.6)	709 (63.8)	
Year of diagnosis			
2011	15 (17.2)	243 (21.9)	0.674 *
2012	15 (17.2)	156 (14.0)	
2013	10 (11.5)	174 (15.7)	
2014	21 (24.1)	176 (15.8)	
2015	14 (16.1)	200 (18.0)	
2016	12 (13.8)	162 (14.6)	
Surgery			
No	62 (71.3)	862 (77.6)	0.176
Yes	25 (28.7)	249 (22.4)	
MCS score			
0–2	54 (62.1)	669 (60.2)	0.716 *
3–5	27 (31.0)	349 (31.4)	
6–8	4 (4.6)	73 (6.6)	
≥9	2 (2.3)	20 (1.8)	

T: trastuzumab; CT: standard chemotherapy; MCS: Multisource Comorbidity Score; * Chi-square test for trend.

**Table 2:** Association between first-line treatment and overall survival in 87 metastatic gastric cancer patients treated with trastuzumab-based chemotherapy (T + CT) and 1111 patients treated with standard chemotherapy (CT).

Variable	*N* (# Deaths)	Hazard Ratio (HR) (95% CI)
Exposure		
CT	1111 (994)	1 ^a^
T + CT	87 (76)	0.73 (0.57–0.93)
Age		
<55	196 (187)	1 ^a^
55–64	297 (284)	1.08 (0.90–1.31)
65–74	362 (363)	1.06 (0.88–1.27)
≥75	251 (236)	1.18 (0.96–1.43)
Sex		
F	708 (377)	1 ^a^
M	398 (693)	1.23 (1.08–1.40)
Year of diagnosis		
2011	258 (235)	1 ^a^
2012	171 (156)	1.04 (0.85–1.28)
2013	184 (174)	1.05 (0.86–1.29)
2014	197 (179)	1.02 (0.83–1.24)
2015	214 (191)	1.08 (0.89–1.31)
2016	174 (135)	0.84 (0.68–1.04)
Surgery		
No	885 (854)	1 ^a^
Yes	251 (216)	0.52 (0.45–0.61)
MCS index		
0–2	682 (631)	1 ^a^
3–5	337 (347)	1.13 (0.98–1.29)
6–8	68 (72)	1.25 (0.97–1.61)
≥9	5 (20)	1.10 (0.70–1.72)

MCS: Multisource Comorbidity Score; ^a^ Reference category.

## Supplementary Materials

The following are available online at https://www.mdpi.com/2072-6694/12/6/1691/s1, Table S1: ICD-9 CM and ATC codes of diseases/conditions and medicament drugs used for the current study.
