# Supplementary material for: Effectiveness and Healthcare Cost of Adding Trastuzumab to Standard Chemotherapy for First-Line Treatment of Metastatic Gastric Cancer: A Population-Based Cohort Study
Source: Cancers (Basel). 2020 Jun 25;12(6):1691. doi: 10.3390/cancers12061691 (PMC7352495; doi:10.3390/cancers12061691)
Supplement: Supplementary file 1 [file cancers-12-01691-s001.pdf]

cancers

MDPI

Supplementary material

Effectiveness and Healthcare Cost of Adding Trastuzumab to Standard Chemotherapy for First-Line Treatment of Metastatic Gastric Cancer: A Population-Based Cohort Study

Table 1. ICD-9 CM and ATC codes of diseases/conditions and medicaments drugs used for the current study.

Disease/Condition	ICD9-CM Codes
Gastric cancer	151.1–151.9
Any malignant cancer	140–208
Chemotherapy	V58.1, 99.25, 99.28
Distant metastasis	197, 198, 199
Surgery	43.42, 43.5, 43.6, 43.7, 43.8, 43.9
**Drug**	**ATC Codes**
Trastuzumab	L01XC03
Any oncologic drug	L01
